# Potential of Essential Oil-Based Anticholinesterase Insecticides against *Anopheles* Vectors: A Review

**DOI:** 10.3390/molecules27207026

**Published:** 2022-10-18

**Authors:** Thankhoe A. Rants’o, Lizette L. Koekemoer, Jenny-Lee Panayides, Robyn L. van Zyl

**Affiliations:** 1 Pharmacology Division, Department of Pharmacy and Pharmacology, Faculty of Health Sciences, University of the Witwatersrand, 7 York Road, Johannesburg 2193, South Africa; 2 WITS Research Institute for Malaria, Faculty of Health Sciences, University of the Witwatersrand, 7 York Road, Johannesburg 2193, South Africa; 3 School of Pathology, Faculty of Health Sciences, University of the Witwatersrand, 7 York Road, Johannesburg 2193, South Africa; 4 Centre for Emerging Zoonotic and Parasitic Diseases, National Institute for Communicable Diseases of the National Health Laboratory Service, 1 Modderfontein Road, Johannesburg 2192, South Africa; 5 Pharmaceutical Technologies, CSIR Future Production: Chemicals, Meiring Naude Road, Pretoria 0184, South Africa

**Keywords:** malaria, insecticides, terpenoids, acetylcholinesterase

## Abstract

The insect nervous system is critical for its functional integrity. The cholinergic system, of which acetylcholinesterase (AChE) is a key enzyme, is essential to the *Anopheles* (consisting of major malaria vector species) nervous system. Furthermore, the nervous system is also the primary target site for insecticides used in malaria vector control programs. Insecticides, incorporated in insecticide-treated nets and used for indoor residual spraying, are a core intervention employed in malaria vector control. However, *Anopheles* resistance against these insecticides has grown rapidly. Due to this major setback, novel agents with potential activity against resistant *Anopheles* and/or capacity to overcome resistance against current WHO-approved insecticides are urgently needed. The essential oils have the potential to be natural sources of novel insecticides with potential to inhibit the *Anopheles* AChE target. In the current review, the scientific evidence highlights the ability of essential oils and specific essential oil constituents to serve as anticholinesterase insecticides. For this reason, the published data from scientific databases on the essential oils and essential oil constituents on anticholinesterase, ovicidal, larvicidal, pupicidal and adulticidal activities were analyzed. The identification of major constituents in active essential oils and their possible influence on the biological activity have also been critically evaluated. Furthermore, the toxicity to mammals as well as potential activity against the mammalian AChE target has also been reviewed. The importance of identifying novel potent insecticides from essential oils has been discussed, in relation to human safety and cost-effectiveness. Finally, the critical insights from this review can be used to inform future researchers towards potent and safe anticholinesterase insecticides for the management of *Anopheles* malaria vectors.

## 1. Introduction

Malaria is a devastating disease caused by a protozoan parasite, namely *Plasmodium falciparum* which is the major causative agent in the pathogenesis of this infectious disease [1,2,3]. *Anopheles* vectors are infected with malaria after they ingest blood from an infected human host. The female *Anopheles* vectors effectively bite the human hosts between dusk and dawn [3] and it is during this time that she ingests gametocytes. The *Plasmodium* gametocytes develop into an oocyst in the mosquito midgut, which then matures into sporozoites. The sporozoites are released into the hemolymph and migrate to the salivary glands [1,4]. This parasite developmental process within the vector takes approximately 11–16 days before the female mosquito is able to transmit the parasite to the next human host during a blood feeding. This means that a long lifespan of the *Anopheles* vector is required for the successful completion of the parasite development and reinfection of the human host. Vertebrate blood is needed every 2–3 days by the female mosquito for nutrition, as well as egg development. The eggs are oviposited into water and fertile eggs hatch into larvae a few days later. Larvae will develop into pupae and finally adults will emerge after a few days [3]. There are more than 400 *Anopheles* species of which about 30 are major malaria vectors. The African *Anopheles* vectors have both long lifespans and a higher preference for human feeding and, collectively, these account for the high malaria cases and mortality that is recorded in Africa [3,5]. Other factors, such as climate conditions and political and economic stability, also affect the intensity of transmission and enhance the problem [3].

The malaria vectors have long been controlled by using insecticides. Insecticide classes include organophosphates, carbamates, pyrethroids, organochlorines and neonicotinoids [6]. Larvicides including insect growth inhibitors as well as bacterial larvicides, such as *Bacillus thuringiensis* subspecies *israelensis, Bacillus sphaericus* and spinosyns from *Saccharopolyspora* species, have also gained popularity in mosquito control activities [6,7,8]. The implementation of large-scale larviciding is however challenging in Sub-Saharan Africa and these may be used as a complementary intervention [6,9,10,11]. The *Anopheles* vectors have developed substantial resistance against almost all current insecticides [12,13,14,15]. To compound the issue, the commercial development of insecticides through various and often complicated synthetic mechanisms is expensive and time-consuming [16,17]. We propose that the identification of potential insecticides from natural product resources, such as essential oils (EOs), is a relatively cost-effective and faster alternative. Target identification and the corresponding mechanism of action are critical components of the drug discovery process [18]. Acetylcholinesterase (AChE) is a validated target in the insect nervous system and inhibitors of this critical enzyme have been useful in the control of malaria vectors for over eighty years [6,19,20].

To facilitate future research in EOs and potential insecticidal activity through AChE inhibition, this review will first discuss malaria vector control systems and current challenges, and we will explore the insect nervous system with relevance to a specific neurotransmitter, acetylcholine (ACh), and provide an in-depth discussion on AChE function and inhibition. Finally, we identify the potential of various EOs and essential oil constituents (EOCs) as anticholinesterase agents against *Anopheles* vectors. This was achieved by collecting scientific data using keywords such as anticholinesterase/acetylcholinesterase inhibition, EOs, terpenoids/terpenes, *Anopheles*, larvicidal, pupicidal, adulticidal and insecticidal in the search engines of PubMed, Google Scholar, ScienceDirect, SciFinder, SCOPUS and Web of Science. 

## 2. Malaria Vector Control

The early vector control strategies adopted vast activities to reduce larval populations, which included, amongst others, the drainage of breeding sites such as swamps or the application of copper (II) acetoarsenite (Paris green), a highly toxic inorganic compound to the breeding sites [21,22]. In addition, the screening of windows and doors to prevent vectors entering houses and the use of mosquito nets have been at the forefront in protecting people against mosquito bites [6]. The plant-based insecticides were the first preparations used historically. Pyrethrins extracted from the flowers of *Chrysanthemum cinerariifolium* and *Chrysanthemum roseum* were used against indoor *Anopheles* mosquitoes in the 19th century [23,24]. However, the structural modifications of the natural pyrethrins and the generation of first synthetic pyrethroids were first reported in the period 1924 to 1970 [25]. The discovery of an organochloride, namely, dichlorodiphenyltrichloroethane (DDT), was reported in 1939 [25,26]. DDT has been highly effective against malaria vectors. However, in recent times increasing safety concerns have seen it being replaced in many countries by newer insecticides with reduced toxicity profiles [27,28,29]. 

Currently, malaria vector control adopts an integrated vector management program through the use of insecticides targeting both the larval and adult stages [6,27]. This is achieved through two main interventions, namely, insecticide-treated mosquito nets and indoor residual spraying, with additional interventions including larviciding [6]. The insecticide-treated nets provide both a physical barrier and insecticidal activity against *Anopheles* vectors. Indoor residual spraying (IRS) on the other hand, provides host protection through the *Anopheles* insecticidal effect [3]. Pyrethroids, pyrethroid-PBO combinations and pyrroles are the only insecticide classes used for the insecticide-treated nets, as the latter insecticides pose a low toxicity risk to humans [30,31]. The pyrethroids used in IRS include deltamethrin, alpha-cypermethrin, etofenprox, lambda-cyhalothrin, bifenthrin and cyfluthrin, while the organochlorines include DDT. On the other hand, the organophosphates approved by WHO for IRS include malathion, fenitrothion, pirimiphos-methyl and the carbamates such as propoxur and bendiocarb [6,32,33,34].

### 2.1. Insecticide Resistance in Main African Malaria Vectors

Insecticide resistance has been reported in all of the main African malaria vectors and this resistance against WHO approved insecticidal agents is rapidly increasing in intensity and geographical distribution [5,35,36]. An overview of mosquito resistance has been highlighted below. To keep this brief, only a few examples will be provided to explain the extent of the problem mainly on the African continent. Insecticide resistance in the main vector species has been reported for pyrethroids [35,37,38,39,40,41], organochlorides [38,41,42,43], organophosphates [12,44] and carbamates [20,38,43,45]. 

Common insecticide resistance markers associated with pyrethroid and organochloride resistance include the L1014F and L1014S mutation of the voltage-gated sodium channel gene, known as knockdown resistance (*kdr*). These mutations shift activation voltage dependence of sodium channels stabilizing them in the closed state. This antagonizes the action of pyrethroids and organochlorines since these compounds bind to open sodium channels [25,41,46,47]. Apart from the *kdr* mutations, elevated metabolic enzymes, including P450 monooxygenases, glutathione-S-transferases and non-specific esterases, also convey high resistance to pyrethroids and organochlorides [13,39,40,44,48,49]. On the other hand, the resistance mechanism commonly conferring organophosphate and carbamate resistance is a single point polymorphism resulting from glycine conversion to a serine residue at position 119 (G119S; *Torpedo californica* AChE numbering) or more precisely, position 280 (G280S; *Anopheles gambiae* AChE numbering) in the AChE target [41,47,50,51]. Resistance mechanisms often prevent the intended biological activity of a specific insecticide; therefore, it is important to discuss modes of action of the major insecticide classes.

### 2.2. Modes of Action of Main Insecticides Used in Malaria Vector Control

Regardless of their small size, insects have a high surface area for the penetration and subsequent systemic distribution of an insecticide from contact exposure. Furthermore, the small size generates short pathways to the insect’s nervous system and as a result, most insecticides act on the insect’s nervous system [52]. Organochlorines act specifically on the peripheral nervous system, where they bind and stabilize the open voltage-gated sodium channels [25]. The stabilized open state of the sodium channels allows for continuous sodium influx and prolonged action potentials leading to spontaneous neuronal firings succeeded by muscle twitches and sustained body tremors [46]. In contrast to the organochlorines, pyrethroids act on both the peripheral and central nervous systems; however, they act in a similar manner to prevent the closing of the voltage-gated sodium channels, resulting in continuous neuronal discharges followed by paralysis [46]. Similarly, the organophosphates and carbamates also exert their effects on the central nervous system. However, these insecticide classes inhibit acetylcholinesterase, a principal enzyme in the insect nervous system, which leads to an increase in the neurotransmitter ACh levels in the synapse. This leads to enhanced ACh effects on the cholinergic receptors resulting in constant neurotransmission and neuronal hyperexcitation [53]. On the other hand, the neonicotinoids enhance cholinergic activity by acting as agonists on nicotinic acetylcholine receptors (nAChRs). Similarly, this disrupts neuronal transmission in the insect nervous system, causing paralysis and subsequent insect death [54]. Since the insect nervous system is the target site for all of the major insecticide classes, it is worth discussing it in more detail.

### 2.3. Insect Nervous System

**Figure 1:**
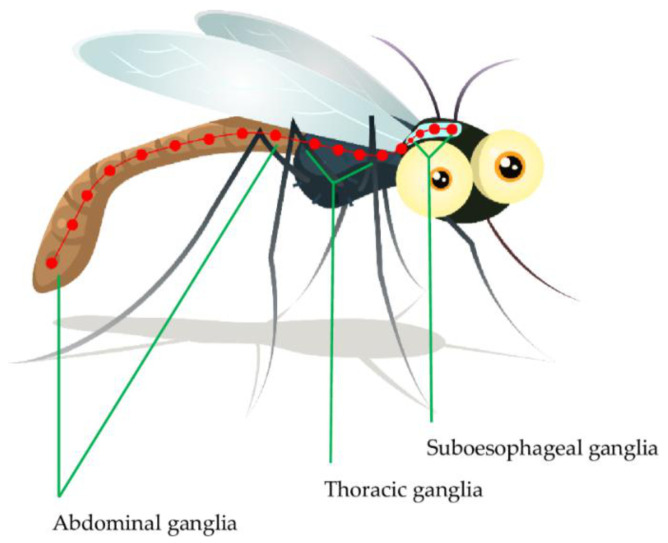
The nervous system ganglia of *Anopheles* [55].

The insect nervous system is composed of central, visceral and peripheral nervous systems [55]. The insect central nervous system (CNS) is composed of the ventral nerve cord and brain connected to various ganglia including supra- and sub-esophageal ganglia, thoracic ganglia and abdominal ganglia (Figure 1). The sub-esophageal ganglion transmits impulses to the mouthparts and salivary glands. The insect brain is composed of three cephalic neuromeres, including the protocerebrum, deutocerebrum and tritocerebrum. The deutocerebrum carries out olfactory and sensory functions through the antennae; where the olfactory signal transduction is important in host identification and interaction by the insect. The tritocerebrum nerves innervate the ventral nerve cord and internal organs including the anterior digestive canal [55,56,57]. The insect’s peripheral nervous system, commonly referred to as a stomatogastric nervous system is composed of the peripheral ganglia complex and nerves that innervate visceral organs. This system mainly controls food intake and digestion. Generally, the insect CNS ganglia receive sensory impulses from the appendages and body cuticle after which the efferent signals are sent to the body muscles, internal organs and genitalia [58,59]. The protocerebrum controls the insect’s vision through compound eyes and ocelli. Most importantly, neurosecretory cells are located in the protocerebrum [59,60]. Most insecticides including the organophosphates and carbamates affect neurotransmitter secretion and action [61,62]. As a result, the insect neurotransmitters, more specifically ACh and its transmission cascade, will be discussed in more detail.

#### 2.3.1. Insect Neurotransmitters

The protocerebrum of the insect brain has nerves that innervate the corpora cardiaca, an organ located posterior to the brain that is composed of the neurohemal and endocrine sections. This enables the corpora cardiaca to perform the important role of neurotransmitter storage and release [55]. Biogenic amines play an important role in the insect nervous system as neuromodulators, neurohormones and neurotransmitters. Moreover, biogenic amines are also a key role player in associative learning and memory for insects. Common neurotransmitters found in the insect CNS include biogenic amines such as dopamine, 5-hydroxy-tryptamine, octopamine, noradrenaline and ACh [58,63,64]. In addition, other neurotransmitters important in the insect nervous system are histamine, glutamate and gamma-aminobutyric acid (GABA). GABA is a main CNS inhibitory neurotransmitter, while glutamate plays an excitatory role both centrally and peripherally [55,65]. For example, the insect heart rate and contraction are regulated by glutamate and other neurotransmitters including ACh, norepinephrine, dopamine, serotonin and octopamine [66]. ACh is a key excitatory neurotransmitter in the insect nervous system [67]. 

#### 2.3.2. Acetylcholine and Its Function in Insects

ACh is synthesized from acetyl-coenzyme A and choline in insect cholinergic neurons and stored in nerve terminals inside presynaptic vesicles from which they are released upon detecting the nerve impulse. This impulse activates voltage-gated calcium channels leading to the influx of calcium ions that then induce ACh release from vesicles via exocytosis [68]. After being released in the synapse, ACh exerts its actions by binding to the postsynaptic nAChRs [69]. ACh is the main excitatory neurotransmitter in insects and its binding to nAChRs and generation of excitatory action potential controls the rapid synaptic neurotransmission process [70]. The nAChRs are ligand-gated channels controlled by binding of ACh and its subsequent removal from the binding site. As long as ACh is bound on the postsynaptic nAChRs, the nerve impulses are transmitted. However, this is short-lived as ACh is rapidly hydrolyzed from the binding site by a specialized enzyme, namely AChE. This ensures that the action potentials are initiated at precise and accurate intervals for efficient neurotransmission [71,72].

#### 2.3.3. Acetylcholinesterase and Its Function in Insects

**Figure 2:**
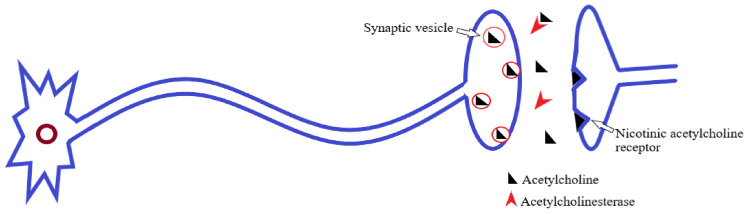
Acetylcholine release, postsynaptic receptor binding, and hydrolysis by acetylcholinesterase [74].

AChE belongs to the general family of cholinesterases; these are the specialized hydrolase enzymes that hydrolyze the choline ester bonds [73]. Therefore, AChE rapidly hydrolyzes the neurotransmitter ACh, to the resultant products, choline and acetate (Figure 2). Through this, it prevents constant nerve firings and maintains normal neuronal impulse transmission at cholinergic synapses and neuromuscular junctions [74,75]. 

Exclusively in insects, the cholinergic system is localized centrally and is absent at the neuromuscular junctions [76]. The cholinergic system is essential for the functioning of the insect nervous system and AChE is a key enzyme in this system [77]. The functional integrity of insects is maintained by its nervous system, and it is for this reason that most insecticides act on the insects’ nervous system [72,78].

### 2.4. Molecular Characterization of Acetylcholinesterase

There are apparent AChE structural differences between insects and mammals. These span from their distinct genomics, amino acid sequences to their active and peripheral anionic site conformations [76]. Recent biochemical studies have revealed critical differences between the *Anopheles* AChE and human AChE that could serve as potential drug targets for directed insecticide design. 

**Figure 3:**
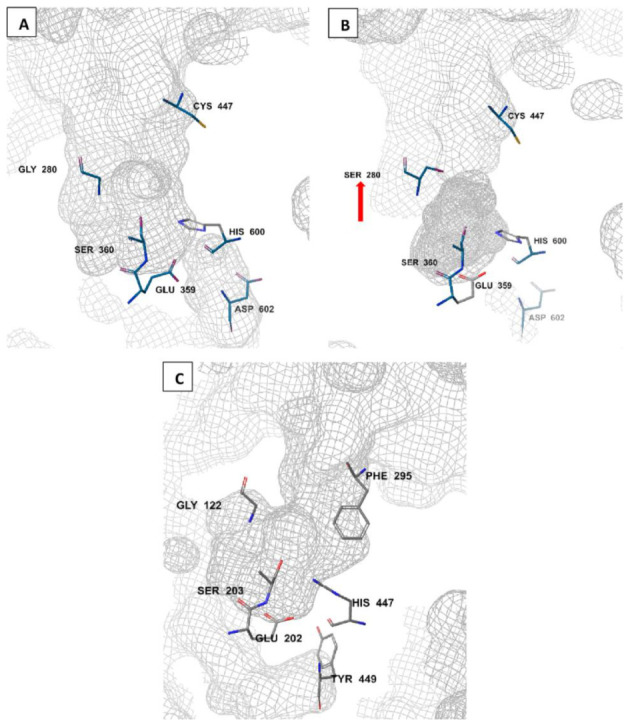
Molecular comparison of *An. gambiae* wild-type (**A**) and resistant (**B**) AChE catalytic sites (PDB IDs: 5YDI and 6ARY, respectively) to the human AChE (PDB ID: 7E3H) (**C**). generated by Schrodinger’s Maestro 2018-2 software (New York, NY, USA). The G280S mutation is shown (red arrow) in the resistant *Anopheles* AChE phenotype (**B**) [51,75].

The amino acid sequence of *Anopheles* AChE is reported to be 48–49% identical to that of the human AChE [79,80]. Unlike humans where there is a single *ace* gene coding for AChE, mosquitoes have two *ace* genes, *ace-*1 and *ace*-2, coding for AChE1 and AChE2 enzymes, respectively [81,82]. These genes are crucial in all life stages of the mosquito, ranging from egg through to adult stages [83]. AChE1 is the main catalytic enzyme, while AChE2 is involved in non-catalytic activities such as reproduction. As a result, target site insensitivity on insect AChEs, such as G280S genotype, is linked to mutations in *ace-*1 but not *ace-*2 [51,62,81]. AChE is characterized by a deep and narrow active-site gorge (Figure 3). There are differences in these gorge structures between *Anopheles* and human AChEs and this may affect ligand binding and specificity [51,79,84]. Notably, a free cysteine residue (Cys^447^) is available at the entrance to the active site gorge of *Anopheles* AChE (Figure 3A,B), but not in human AChE. Instead, a human AChE has a bulky phenylalanine (Phe^295^) at the active site entrance (Figure 3C). Additionally, in *Anopheles* AChE, a smaller aspartic acid residue (Asp^602^; Figure 3A,B) replaces a larger tyrosine residue (Tyr^449^) at the base of the active site gorge [51]. Moreover, a conserved arginine residue (Arg^339^; not shown in order to maintain the catalytic side resolution) has also been identified in *Anopheles* AChE [85]. In addition, the displayed *An. gambiae* AChE catalytic site in Figure 3B shows a G280S mutated site (pointed).

#### 2.4.1. Acetylcholinesterase Inhibition in Anopheles

The catalytic site in *Anopheles* is characterized with a catalytic triad made of His-Ser-Glu (His^600^-Ser^360^-Glu^359^; Figure 3A) amino acid combination. The catalytic serine (Ser^360^; Figure 3A) is the target for covalent insecticides, including organophosphates and carbamates [61,62]. These insecticides establish a covalent bond with AChE through phosphorylation and carbamoylation, respectively [67]. The inhibition of AChE leads to ACh accumulation that eventually results in over-stimulation of postsynaptic cholinergic receptors [76]. This neuroexcitation causes rapid insect paralysis and death [72,76]. The *Anopheles* resistance to the anticholinesterase insecticide classes is usually caused by *ace-*1 G280S mutation (Figure 3B) and metabolic resistance resulting from the elevated levels of monooxygenases, glutathione-S-transferases and general esterases [62,86,87,88]. Given the widespread resistance that has largely rendered organophosphates and carbamates non-effective, there is an urgent need to identify novel anticholinesterase insecticides. AChE has proven to be a valid target in *Anopheles* vectors [79] and EOs have also shown to be the promising sources of novel insecticides [89,90,91]. Interestingly, the EOs have shown activity against resistant *Anopheles* species and a capability to synergize conventional insecticides including the pyrethroids [92,93]. Additionally, the EOs and their constituents inhibit the P450 monooxygenase and glutathione-S-transferase detoxification enzymes involved in multiple insecticide resistance including the pyrethroids, organophosphates and carbamates. The insect toxicity of pyrethroids, organophosphates and carbamates has been greatly enhanced by synergy with EOs including cedarwood oil, geranium oil, clove oil, patchouli oil, cinnamon oil, basil oil, oregano oil, purple nutsedge oil, thyme, coriander and galangal oil [94,95,96]. The identified individual EOCs capable of synergizing conventional insecticides, especially pyrethroids, include thymol, eugenol, carvacrol, geraniol and linalool [96,97]. 

##### Essential Oils and Constituents as Potential Anopheles AChE Inhibitors

EOs are hydrophobic secondary metabolites extracted from different aromatic plant parts by steam distillation, hydro-distillation, head-space analysis, solvent extraction or liquid carbon dioxide extraction [93,98]. Various terpenoids including sesquiterpenes, monoterpenes, diterpenes and phenylpropanoids are major phytoconstituents in EOs [99,100]. Many EOs and certain EOCs have exhibited significant anticholinesterase activity in in vitro studies. In insects, many EOs have been reported to exhibit neurotoxic effects characterized by rapid paralysis and death [98], which links them to AChE inhibition. Interestingly, most EOCs can only inhibit mammalian AChE very weakly. In addition, most EOs and EOCs are relatively non-toxic to mammals with very low potency for acute oral toxicity, whereby lethal doses for pure compounds range from 800 to 3000 mg/kg and more than 5000 mg/kg for EOs or EOCs incorporated in pharmaceutical formulations [93,101,102]. The EOs are natural resources and therefore, the identification of novel insecticides from such sources is relatively cost-effective. Production of insecticides from EOs can be relatively less expensive translating into low costs for the malaria endemic countries [103,104]. In addition, the discovery of insecticides is relatively rapid since the lead compounds are screened on the actual insect as opposed to pharmaceuticals intended for human use that undergo a lengthy translational science process from the in vitro to the in vivo studies including animal models and human clinical trials [105].

In vitro anticholinesterase activity is usually assessed with the Ellman assay, a globally accepted method to assess the AChE activity and potential inhibition thereof [106]. For such studies, the insect homogenate may be used as a crude enzyme source [107,108,109]. However, some studies use AChE from *Electrophorus electricus* (electric eel) to estimate insect AChE activity [110,111]. The electric eel and *Anopheles* AChEs are reported to have nearly the same backbone conformation [85]. For the purpose of this review, EOs or EOCs inhibiting AChE in the in vitro studies are only considered to have potential *Anopheles* anticholinesterase activity if they have shown *Anopheles* insecticidal activity in addition to the observed AChE inhibition. Furthermore, where available, the selectivity between human and *Anopheles* AChE targets, as well as human toxicity potential for the EOs and EOCs, are extensively discussed.

##### The EOs as Potential Anticholinesterase Insecticides

Several EOs have shown insecticidal activity against *Anopheles* species [93]. Given their high volatility, the EOs can reach their target through inhalation by the insect species, ingestion or contact [112]. The absorption of EOs is facilitated by the lipophilic character and low molecular weights of their active constituents [100,112]. This behavior is also important for the partitioning of EOs into the insect midgut plasma membrane [112,113].

Some of the EOs have not only shown the insecticidal effects, but also exhibited anticholinesterase activity indicating a potential to be anticholinesterase insecticides [102]. With increasing resistance against current insecticides, the potential use of EOs as alternative insecticides has been suggested [93]. Various EOs have shown potential to be active against resistant colonies including pyrethroid resistant *Anopheles* species, such as *An. gambiae* and *An. stephensi* [93,114,115]. Furthermore, the EOs and EOCs have shown potential to synergize with conventional insecticides and to inhibit those enzymes responsible for insecticide detoxification, which shows promise in overcoming resistance [88,92,94]. This study identified essential oils from 16 plant species with anticholinesterase potential and corresponding insecticidal capabilities (Table 1). Seven of these exhibited both anticholinesterase and insecticidal activities at IC_50_ and LC_50_ values less than 100 µg/mL, indicating high potencies [116]. These highly potent EOs with potential of being anticholinesterase insecticides include *Hyptis suaveolens*, *Hyptis spicigera*, *Ocimum canum*, *Lantana camara*, *Ferulago carduchorum*, *Ferulago trifida*, *Salvia officinalis* and *Curcuma longa* (Table 1); the latter EOs belong to the general plant families of Lamiaceae, Verbenaceae, Apiaceae and Zingiberaceae [93,117,118].

Notably, one EO could exhibit considerable differences in anticholinesterase and/or insecticidal activity (Table 1). This is probably due to variations in the identity or quantity of its specific phytoconstituents. Three *Salvia officinalis* EOs from different locations in Italy exhibited anticholinesterase activity at IC_50_ values of 47.68, 58.35 and 77.51 µg/mL [135]. While these EOs had a similar content of camphor (16.84%, 16.16% and 18.92%, respectively) as the main constituent, it was notable that the borneol content in the third EO (IC50: 77.51 µg/mL) was approximately half (2.34%) of that yielded from the former EOs (4.48% and 4.68%, respectively) [135]. Borneol has been previously shown to inhibit AChE [121]. Using similar experimental conditions and AChE enzyme concentrations, the *Ocimum canum* Sims. EOs from Burkina Faso inhibited AChE at the IC_50_ value of 0.21 µg/mL [110] and a relatively higher value of 36.16 µg/mL [133]. The latter EO had 59.9% content of 1,8-cineole as the main constituent, while the composition of the former and more active EO was not mentioned [110,133]. *Mentha pulegium L.* EO from Iran exhibited the larvicidal activity against *An. stephensi* with the LC_50_ value of 40.13 µg/mL [130], while that from Portugal attained the LC_50_ of 113.6 µg/mL against the same *Anopheles* species [132]. *Mentha pulegium* L. EO from Portugal had 61.4% pulegone and 20% menthone as the main constituents, while the phytochemical analysis was not reported for the *Mentha* species from Iran [130,132]. 

Major constituents in EOs are often responsible for the observed biological activity associated with such a sample [100,140,141]. For this reason, this review collected phytochemical data on the major components in the identified bioactive EOs (Table 2). Some of these major constituents possess anticholinesterase and insecticidal activity as displayed in Table 3. Common constituents in most EOs include α-pinene, β-pinene, p-cymene, γ-terpinene and β-caryophyllene. Certain EOCs such as terpinen-4-ol, 1,8-cineole, menthone, menthol, fenchone, γ-terpinene, (-)-bornyl acetate, linalool, citral and pulegone have been reported as competitive AChE inhibitors [98,142,143,144]. Additionally, common constituents in seven of the most active EOs, especially *Hyptis suaveolens*, *Hyptis spicigera*, *Ocimum canum*, *Lantana camara*, *Ferulago carduchorum*, *Ferulago trifida* and *Salvia officinalis* are α-pinene, β-pinene, β-caryophyllene and γ-terpinene (Table 2). The *Curcuma longa* EO has a unique composition profile of ar-turmerone, tumerone, curlone, α-curcumene and β-sesquiphellandrene [123]. The main constituent ar-turmerone has only attained anticholinesterase activity against human AChE with an IC_50_ value of >100 μg/mL (IC_50_: 191.1 ± 0.3 μg/mL), indicating a low potency [116]. *Curcuma longa* EO has been reported as possessing an anticholinesterase with an IC_50_ value of 34.70 ± 3.10 μg/mL. The *Curcuma longa* EO has also been reported to possess strong larvicidal activity with the LC_50_ range of 1.5 to 34 μg/mL [122,123], indicating that it may be selectively targeting the insect. 

##### The EOCs as Potential Anticholinesterase Insecticides

Some studies have isolated the active principles from EOs and demonstrated the potential of these to exert *Anopheles* anticholinesterase activity [98,156]. Moreover, some of these have also exerted *Anopheles* mortality in insecticide susceptibility assays [93,131,157]. Gnankiné and Bassolé (2017) reported that several constituents in EOs possess ovicidal, larvicidal and adulticidal effects against *Anopheles* species. In this study, 22 EOCs from various terpenoid classes such as sesquiterpene alcohols, sesquiterpene oxides, monoterpenoids and monoterpene alcohols, as well as phenylpropanoids, have been identified as possessing both anticholinesterase and insecticidal activity against *Anopheles* species (Table 3). Some of these EOCs, such as 1,8-cineole, α-pinene, camphor, linalool, borneol, (+)-3-δ-carene, γ-terpinene, caryophyllene oxide, *p*-cymene, (*E*)-anethole, terpinen-4-ol, pulegone and limonene, have shown low potency towards human AChE [98,102,158,159,160].

The α-pinene, estragole, carvacrol, (+)-δ-3-carene, eugenol and camphor were the most active in terms of both AChE inhibition and insecticidal activity (Table 3); thus, indicating that these EOCs show potential to serve as anticholinesterase insecticides. Interestingly, previous studies have shown that these EOCs have a lower activity against human AChE. For example, camphor and α-pinene could only inhibit AChE from human erythrocytes with IC_50_ values of >10 mM and 0.4–0.7 mM, respectively [159,161]. Similarly, an IC_50_ range of 0.2 to 0.3 mM of (+)-3-δ-carene was needed to inhibit bovine and human erythrocytes AChE [159,161]. These EOCs may therefore produce insecticides with high selectivity towards *Anopheles* AChE inhibition over the human target.

*Monoterpenoids:* The monoterpenoid α-pinene is the main constituent in many essential oils [93]. Orhan et al. (2008) reported that α-pinene, but not β-pinene, has anticholinesterase properties [162]. α-Pinene has shown anticholinesterase activity at an IC_50_ value as low as 22 μg/mL [163]; however, this was higher than its parent EOs from *Hyptis suaveolens* and *Hyptis spicigera* that obtained IC_50_ values between 0.5 and 6.5 μg/mL [110]. Apart from the fact that different *Anopheles* species were used, this suggests the possibility of synergism among the EOCs in such EOs. Due to their smaller molecular weights, more than one monoterpenoid can bind to the AChE catalytic and/or peripheral site and, usually, binding of one monoterpenoid facilitates binding of the other [102]. On the other hand, α-pinene exhibited larvicidal activity (LC_50_ of 32.1 μg/mL) against *An. subpictus* that was comparable to its anticholinesterase activity [93,163]. In another study, Wojtunik-Kulesza et al. (2017) reported a higher anticholinesterase IC_50_ value of 102.0 mM for α-pinene along with declaration that insolubility of the EOCs affected the accuracy of spectrophotometric measurements [164]. Generally, the observed variations were caused by the use of different AChE types and protein contents. For example, using 0.25 U/mL AChE, Farag et al. (2016) determined an IC_50_ of 0.337 µM for estragole; meanwhile, Lopez et al. (2015) doubled the AChE concentration and obtained an IC_50_ value of 12.6 mM [102,165]. The purity and stability of reagents and EOs or EOCs used in AChE activity assays may also affect the outcome [166,167]. Additionally, AChE activity is a pH-dependent reaction [168] and variations in pH across studies may cause potential differences in inhibition kinetics. 

A major constituent in the EO of *Echinophora lamondiana*, (+)-δ-3-carene, resulted in comparable anticholinesterase (IC_50_ value: 36 µg/mL) and larvicidal (LC_50_ value of 42.9 µg/mL) activity against *An. quadrimaculatus* [169,170]. The latter larvicidal LC_50_ value of the EOC was observed to be similar to that obtained for the EO of the flowers (LC_50_: 46.9 µg/mL) with 61.9% (+)-δ-3-carene content, but higher than the EOC of the leaves (LC_50_: 26.2 µg/mL) with comparable (+)-δ-3-carene content (75.0%) [169]. Though less predominant than (+)-δ-3-carene in the *Echinophora lamondiana* EO, both terpinolene (2.7 to 3.3%) and α-phellandrene (12.8 to 20.3%) were reported to be even more potent larvicidal agents than (+)-δ-3-carene, with LC_50_ values of 20.9 and 15.6 µg/mL, respectively [169]. 

Camphor, a monoterpenoid ketone and the major component in the EOs of *Ocimum africanum* and *Ocimum americanum*, was also shown to exhibit low anticholinesterase activity (IC_50_ value of 21.43 µM) [165]. Moreover, camphor is known to non-competitively inhibit nAChRs [98,102,171]. Camphor is also active as a repellent against *An. culicifacies, An. gambiae* and *An. funestus* [172,173,174]. However, poor larvicidal activity by camphor was obtained with a LC_50_ value > 100 µg/mL [175]. 

Aazza et al. (2011) reported that the EO of *Thymus vulgaris* consists of 16% carvacrol. Interestingly, carvacrol is more than three times as active (IC_50_ value of 63.0 μg/mL) as its parent EO (IC_50_ value of 216.9 μg/mL) in AChE inhibition studies [121,139]. This indicates the potential effect of antagonistic interactions within the complex mixture of the *Thymus vulgaris* EO resulting in decreased activity. However, the *Anopheles* anticholinesterase potential of carvacrol (IC_50_ value of 63.0 μg/mL) is non-specific as its activity is comparable to that against AChE from bovine serum (IC_50_ value of 70.3 μg/mL) [139,162]. Stereochemistry in AChE inhibition also plays a role, whereby carvone (a monoterpenoid ketone) attained weak AChE inhibition (IC_50_ value of 830 μg/mL), in comparison to the structurally related phenolic monoterpenoid, carvacrol (IC_50_ value of 63.0 μg/mL) [93,139,164]. Carvone has previously been reported to be a potent non-competitive AChE inhibitor of mammal AChE [102,162].

Another phenolic monoterpene, thymol, is ineffective in inhibiting AChE, but is a positive allosteric modulator of the insect’s GABA-A receptors [118,164]. Thymol has also been reported to act at the octopamine receptors; this is where octopamine is an analogue of norepinephrine, functioning as a neurotransmitter, neuromodulator and neurohormone in insects [176]. Most studies have shown thymol to be as ineffective as its parent EO *Thymus vulgaris* against the AChE target [121,139].

*Phenylpropanoids:* Estragole is a phenylpropanoid isolated from the EOs of the *Ocimum* species. Along with strong anticholinesterase activity (IC_50_ value of 0.337 µM) [165], it has shown similarly potent larvicidal activity against *An. stephensi* (LC_50_ value of 11.01 μg/mL) and *An. atroparvus* (LC_50_ value of 15.7 μg/mL) [102,118]. Estragole was reported to be potentially selective for *Anopheles*, as it has shown no inhibition of mammal AChE [162]. 

Eugenol, an allyl chain-substituted guaiacol (phenol), is a major constituent (31.12%) in the *Plectranthus barbatus* EO, an EO with the LC_50_ value of 84.2 µg/mL, against *An. subpictus* larvae [177]. However, eugenol is three times more potent (LC_50_ value of 25.45 µg/mL) than this latter parent EO as a larvicide against *An. subpictus* [93]. Again, this suggests possible antagonism with other constituents. Eugenol and (*E*)-anethole (a phenylpropanoid) reportedly interacted in an antagonistic manner when tested for larvicidal activity [178]. Although eugenol had an IC_50_ value of 40.32 µg/mL for AChE inhibition, this inhibitory activity was however comparable to that attained against bovine erythrocytes AChE (42.44 ± 1.21 µg/mL) [165,179]. In contrast, Dohi et al. (2009) reported a much higher IC_50_ value for eugenol (480 µg/mL) against electric eel AChE [163]. Based on these two findings, eugenol has a potential nonselective AChE inhibition, and to be an antagonist at insect octopamine receptors [93]. 

While showing potent insecticidal activity, some EOs and EOCs did not show any evidence that their mode of action involved targeting AChE. Pulegone has a potent insecticidal activity against *An. stephensi*, however, it could not efficiently inhibit AChE [141,164]. In contrast, the epoxide form of pulegone, the pulegone-1,2-epoxide isolated from the *Lippia steochadifolia* EO, has be reported to be an insect neurotoxin, acting as an irreversible inhibitor of AChE [191]. In addition, the *Citrus limon* EO and it major EOC, limonene (99%) both possess weak AChE inhibition properties; whilst both are potent larvicides against *An. gambiae* and *An. stephensi* [120,121,184]. This suggests that a different mode of action may be responsible for their insecticidal activity. In *Cimex cimicidae* (bedbugs), limonene has been proposed as acting by destroying the wax layer of the insect respiratory system [192]. 

This review identified EOs and specific EOCs with anticholinesterase activity and indeed the capacity to cause *Anopheles* mortality. The EOs and EOCs spectrum of activity against *Anopheles* included ovicidal, larvicidal and adulticidal activities, where they were regarded as active if they obtained LC_50_ values less than 100 µg/mL [193]. The common *Anopheles* species involved in global malaria transmission that have been assessed include *An. stephensi*, *An. gambiae*, *An. arabiensis*, *An. subpictus*, *An. anthropophagus*, *An. quadrimaculatus*, *An. dirus*, *An. cracens*, *An.labranchiae*, *An. sinensis* and *An. atroparvus* [194,195]. Interestingly, almost all of these have already developed clinically significant resistance against current insecticides [194,196,197,198]. The commonly assessed *An. Stephensi*, that was susceptible to many of the EOs and EOCs, is predominant in Asia and recently invaded Africa [199,200]. This species is resistant to conventional AChE insecticides, including organophosphates and carbamates, and has also shown resistance to other insecticide classes such as pyrethroids and organochlorines [201,202]. The EO of *Ferulago carduchorum* has shown both larvicidal and anticholinesterase activity at LC_50_ and IC_50_ values around 12 µg/mL, which are extremely promising [126]. 

Interestingly, the EOs of *Hyptis suaveolens*, *Hyptis spicigera*, *Ocimum canum* and *Lantana camara* have shown activity against all three developmental stages of *An. gambiae*, a main African vector. Meanwhile, the EO of *Mentha pulegium* and *Ocimum canum* have been shown to target the larval stage of *An. gambiae* [93,131] and *An. funestus* (LC_50_ value of 91.2 µg/mL), respectively [93,195,203]. Of all the species, *An. arabiensis* is the main vector in Sub-Saharan Africa [48] against which the EOs of *Schinus mole* and *Eucalyptus globulus* have displayed larvicidal activity with LC_50_ values less than 100 µg/mL. However, the anticholinesterase activity of these latter EOs, as well as their major EOCs, α-phellandrene and eucalyptol, respectively, were in the millimolar range indicating that their larvicidal activity is not primarily through AChE inhibition. Both *Schinus mole* and *Eucalyptus globulus* EOs are non-toxic towards mammals and the *Eucalyptus globulus* EO is recommended for skin application as a repellent [93,204]. 

It is crucial to identify novel insecticides from EOs as most are relatively safe to mammals [93,118]. An EO of *Foeniculum vulgare* could only exhibit acute oral toxicity at the LD_50_ of 3120 mg/kg in the rat model [118]. This EO is active against *An. stephensi* and *An. dirus* with LD_50_ values between 20 and 35 µg/mL, thus indicating a high safety index for human use [93]. The EOCs, (*E*)-anethole, eugenol, limonene, thymol and γ-terpinene, displayed acute oral toxicity at the LD_50_ values ranging from 980 to 4600 mg/kg [118] where all attained insecticidal LC_50_ values less than 100 µg/mL (Table 3); thus, indicating their potential as bioinsecticides. 

## 3. Conclusions and Future Perspective

The *Anopheles* vectors are responsible for malaria transmission across the world; additionally, most of these vectors have acquired resistance against current insecticide classes. *Anopheles* AChE is a valid target by conventional AChE inhibitors in the market. Due to the current resistance status towards anticholinesterase insecticides, organophosphates and carbamates, the identification of novel insecticides is critical. The EOs and EOCs have shown potential to serve as anticholinesterase insecticides against *Anopheles* vectors. This is afforded by the capability of some EOs and EOCs to exhibit both anticholinesterase and insecticidal activity. In this review, seven EOs from *Hyptis suaveolens*, *Hyptis spicigera*, *Ocimum canum*, *Lantana camara*, *Ferulago carduchorum, Ferulago trifida*, *Salvia officinalis* and *Curcuma longa* plant species which showed potent anticholinesterase and insecticidal activities against various *Anopheles* species were summarized. Along with these, six EOCs, namely, α-pinene, estragole, carvacrol, (+)-3-δ-carene, eugenol and camphor, were identified as being the most active against the *Anopheles* vector and AChE target. All of these, except for eugenol and carvacrol, have the potential to be selective towards *Anopheles* AChE. This scientific data review is important for informing future research towards novel anticholinesterase insecticides for malaria vector control. Future studies in this area should focus on the EOs and EOCs identified in this review for possible development into insecticidal agents. Active constituents should be identified from the EO complex mixtures and possible synergistic interaction taken into consideration. The EOs and the identified EOCs in this review require further assessment against various stages of the *Anopheles* life cycles and potential activity against insecticide resistant *Anopheles* vectors. Moreover, these promising EOs and EOCs should be assessed for possible activity against human AChE, toxicity against other aquatic lives, as well as for mammal toxicity.

In general, this review supports the future development of EOs as a potential source for novel insecticides with AChE inhibitory potential; including the specific EOs with potential anticholinesterase and insecticidal activities outlined in this review, belonging to the families of Lamiaceae, Verbenaceae, Apiaceae and Zingiberaceae. Meanwhile, the promising EOCs for development into novel anticholinesterase insecticides belong to broader classes of sesquiterpene alcohols, monoterpenoids, monoterpene alcohols and phenylpropanoids.

## Figures and Tables

**Table 1:** Anticholinesterase and insecticidal activities of EOs.

Essential Oil	Anticholinesterase IC_50_	Insecticidal Activity	Insecticidal LD_50_	*Anopheles* Species (Laboratory Strain)	Reference(s)
*Carum carvi*	0.82 ± 0.05 mg/mL	Larvicidal	72.00 μg/mL	*An. dirus*	[93,119]
*Citrus limon*	0.85 ± 0.01 mg/mL	Larvicidal	13.75 μg/mL	*An. gambiae (Kisumu)*	[120,121]
			32.28 μg/mL	*An. gambiae Logbessou*	
*Curcuma longa*	34.70 ± 3.10 μg/mL	Larvicidal	33.61 μg/mL	*An. cracens*	[122,123,124]
		Larvicidal	1.8–3.7 μg/mL	*An. quadrimaculatus*	
*Eucalyptus globulus* Labill.	0.13 ± 0.01 mg/mL	Larvicidal	0.09 mg/mL	*An. arabiensis*	[121,125]
*Ferulago carduchorum*	23.6 µg/mL	Larvicidal	12.00–12.78 µg/mL	*An. stephensi*	[93,126]
*Ferulago trifida*	21.5 ± 2.2 mg/mL	Larvicidal	10.46 µg/mL	*An. stephensi*	[127]
*Foeniculum vulgare* Mill.	1.19 ± 0.01 mg/mL	Larvicidal	35.30 μg/mL	*An. dirus*	[93,121,128]
		Larvicidal	20.10 μg/mL	*An. stephensi*	
*Hyptis* *spicigera* Lam.	6.3 ± 0.43 µg/mL	Ovicidal Larvicidal Adulticidal	69.61 µg/mL 45.18 µg/mL 1.45% *w*/*v*	* An. gambiae	[110,129]
		Adulticidal	1.04% *w*/*v*	*An. gambiae* (Kisumu)	
*Hyptis* *suaveolens* Poit.	0.55 ± 0.12 µg/mL	Ovicidal Larvicidal Adulticidal	61.71 µg/mL 159.5 µg/mL 1.86% *w*/*v*	* An. gambiae	[110,129]
		Adulticidal	0.85% *w*/*v*	*An. gambiae* (Kisumu)	
*Lantana camara* L.	1.75 ± 0.12 µg/mL	Ovicidal Larvicidal Adulticidal	53.59 µg/mL 61.69 µg/mL 0.84% *w*/*v*	* An. gambiae	[110,129]
		Adulticidal	0.24% *w*/*v*	*An. gambiae* (Kisumu)	
*Mentha pulegium* L.	108.75 µg/mL	Larvicidal	40.13–113.6 µg/mL	*An. stephensi*	[130,131,132]
		Larvicidal	118.0 µg/mL	*An. gambiae*	
		Larvicidal	58.9 µg/mL	*An. atroparvus*	
*Ocimum canum* Sims.	0.21–26.16 µg/mL	Ovicidal Larvicidal Adulticidal	71.56 µg/mL 138.7 µg/mL 1.84% *w*/*v*	* An. gambiae	[93,110,129,133]
		Adulticidal	1.22% *w*/*v*	*An. gambiae* (Kisumu)	
		Larvicidal	74.12 µg/mL	*An. funestus*	
*Salvia leucantha*	>250 µg/mL	Larvicidal	10.9 µg/mL	*An. quadrimaculatus*	[93,134]
*Salvia officinalis*	47.68–77.51 µg/mL	Larvicidal	14.1 µg/mL	*An. quadrimaculatus*	[93,135]
* Schinus molle * L.	6.54 ± 0.15 mg/mL	Larvicidal	21.0 µg/mL	*An. arabiensis*	[136,137]
*Thymus vulgaris*	0.22–0.54 mg/mL	Larvicidal	351.63 μg/mL	*An. labranchiae*	[121,138,139]

* Wild population.

**Table 2:** Major constituents of EOs with anticholinesterase insecticidal activity.

Essential Oil	Major Constituents	References
*Carum carvi*	γ-Terpinene, β-pinene, bornyl acetate, carvone, *p*-cymene	[119]
*Citrus limon*	Limonene	[121]
*Curcuma longa*	ar-Turmerone, tumerone, curlone, α-curcumene, β-sesquiphellandrene	[123]
*Eucalyptus globulus* Labill.	Eucalyptol, α- pinene, *p*-cymene, β-cymene, 1,8-cineole, limonene	[121,145,146]
*Ferulago carduchorum*	(*Z*)-β-ocimene, α-pinene, bornyl acetate	[126]
*Ferulago trifida*	Isobornyl acetate, trans-verbenol, (*E*)- β-caryophyllene	[127]
*Foeniculum vulgare* Mill.	Thymol, estragole, α-phellandrene, limonene, (*E*)-anethole, fenchone,	[121,123,147]
*Hyptis* *spicigera* Lam.	β-caryophyllene, α-pinene, β-pinene, α-phellandrene, α-thujene, sabinene	[129,148,149]
*Hyptis* *suaveolens* Poit.	1,8-cineole, sabinene, terpinolene α-thujene, α- pinene, β-pinene	[129,150,151]
*Lantana camara* L.	β-caryophyllene, α-humulene, γ-curcumene, germacrene D	[152,153]
*Mentha pulegium* L.	Pulegone, neomenthol, menthone	[131,132]
*Ocimum canum* Sims.	Thymol, *p*-cymene, γ-terpinene, estragole, linalool	[154,155]
*Salvia leucantha*	Bornyl acetate, 6,9-guaiadiene, (*E*)-β-caryophyllene, bicyclogermacrene, camphene, α- pinene, β-pinene	[117,134]
*Salvia officinalis*	Camphor, camphene, α-thujone, 1,8-cineole, α- pinene, β-pinene	[135]
*Schinus molle* L.	α-Phellandrene, β-phellandrene, α- pinene, β-pinene, γ-terpinene, *p*-cymene	[136]
*Thymus vulgaris*	*p*-Cymene, borneol, thymol, carvacrol	[121]

**Table 3:** Anticholinesterase and insecticidal activities of EOCs.

Essential Oil Constituent	Anticholinesterase IC_50_	Insecticidal Activity	Insecticidal LD_50_	*Anopheles* Species (Laboratory Strain)	Reference(s)
*(E)-*Anethole	1.324 ± 0.011 mg/mL	Larvicidal	25.11 μg/mL	* An. sinensis	[121,178]
Borneol	0.132 ± 0.012 mg/mL	Larvicidal	35.89 μg/mL	*An. anthropophagus*	[121,180]
Camphor	0.003–1.7 mg/mL	Larvicidal	129.17 μg/mL	*An. anthropophagus*	[102,165,175]
(+)-δ-3-Carene	0.036–0.64 mg/mL	Larvicidal	42.9 µg/mL	*An. quadrimaculatus*	[169,170]
Carvacrol	0.063–0.092 mg/mL	Larvicidal	21.1–24.06 µg/mL	*An. subpictus*	[121,139,181]
		Larvicidal	21.15 µg/mL	*An. stephensi*	
Carvone	0.437–0.83 mg/mL	Larvicidal	19.3 μg/mL	*An. stephensi*	[102,157,164]
Caryophyllene oxide	>200 µg/mL	Larvicidal	49.46 µg/mL	*An. anthropophagus*	[93,175,182]
Estragole	0.337 µM–12.6 mM	Larvicidal	15.7 μg/mL	*An. atroparvus*	[102,118,165]
			11.01 μg/mL	*An. stephensi*	
Eucalyptol	266.0 ± 11.87 mM	Larvicidal	>200 µg/mL	*An. anthropophagus*	[93,102,164]
Eugenol	40–480 mg/mL	Larvicidal	93.14 µg/mL	*An. stephensi*	[163,165,178,181,183]
			25.45 µg/mL	*An. subpictus*	
			31.09 µg/mL	* An. sinensis	
Isopulegol	233.0 ± 10.08 mM	Larvicidal	49.4 μg/mL	*An. gambiae s.s*	[93,102]
Limonene	220–586 μg/mL	Larvicidal	8.8 μg/mL	*An. stephensi*	[121,184,185,186]
		Larvicidal	18.91 μg/mL	*An. sinensis*	
Linalool	1.69–2.4 mg/mL	Larvicidal	35.87 μg/mL	*An. anthropophagus*	[102,164,180]
*(Z*)-*β*-Ocimene	4.7 ± 0.20 mM	Larvicidal	25.84 μg/mL	*An. stephensi*	[118,164]
			30.86 μg/mL	*An. subpictus*	
α-Phellandrene	0.12–3.68 mg/mL	Larvicidal	15.6 μg/mL	*An. quadrimaculatus*	[102,169]
*α*-Pinene	0.022–1.43 mg/mL	Larvicidal	32.1 μg/mL	*An. subpictus*	[163,164,165,187]
Pulegone	9.0 ± 0.41 mM	Larvicidal	48.9 μg/mL	*An. stephensi*	[141,164]
Sabinene	176.5 ± 2.8 μg/mL	Larvicidal	19.67 μg/mL	*An. stephensi*	[118]
Terpinen-4-ol	0.19–3.2 mg/mL	Larvicidal	47.73 μg/mL	*An. subpictus*	[143,156,163,186,188]
		Larvicidal	43.27 μg/mL	*An. stephensi*	
		Larvicidal	62.09 μg/mL	*An. sinensis*	
		Larvicidal	337.7 μg/mL	*An. gambiae s.s*	
γ-Terpinene	5.8 mM	Larvicidal	44.61 μg/mL	*An. anthropophagus*	[102,180,186]
		Larvicidal	36.42 μg/mL	*An. sinensis*	
α-Terpineol	1.3 ± 0.06 mg/mL	Larvicidal	39.98 μg/mL	*An. sinensis*	[163,186]
Terpinolene	156.4–550.0 µg/mL	Larvicidal	404.71 µg/mL	*An. gambiae s.s*	[93,118,156,169,170]
		Larvicidal	20.9–25.7 µg/mL	*An. quadrimaculatus*	
Thymol	0.05–0.74 mg/mL	Larvicidal	10.3–22.06 μg/mL	* An. subpictus *	[118,139,141,189,190]
		Larvicidal	48.88 μg/mL	*An. stephensi*	

* Wild population.

## Data Availability

Publicly available molecular datasets were analyzed in this review. The data can be found in Protein Data Bank (https://www.rcsb.org/ accessed on 1 September 2022): [PDB IDs: 5YDI, 6ARY and 7E3H].
